# Anti-inflammatory effects of palm^11^-PrRP31 in a rat model of lipopolysaccharide-induced acute inflammation

**DOI:** 10.1530/JME-24-0090

**Published:** 2025-02-14

**Authors:** Lucia Mráziková, Silvie Hojná, Anna Shánělová, Blanka Železná, Jaroslav Kuneš, Lenka Maletínská

**Affiliations:** ^1^ Institute of Organic Chemistry and Biochemistry, Czech Academy of Sciences, Prague, Czech Republic; ^2^ Institute of Physiology, Czech Academy of Sciences, Prague, Czech Republic; ^3^ First Faculty of Medicine, Charles University, Prague, Czech Republic

**Keywords:** palm^11^-PrRP31, inflammation, lipopolysaccharide, cytokines, chemokines

## Abstract

Lipopolysaccharides (LPS) are major components of gram-negative bacteria. LPS not only induce endotoxemia and inflammation but also contribute to various diseases. In experimental settings, LPS administration serves as a model for acute inflammatory responses. This study aimed to evaluate the anti-inflammatory potential and mechanism of action of palmitoylated prolactin-releasing peptide (palm^11^-PrRP31) in a rat model of LPS-induced inflammation. Palm^11^-PrRP31 has demonstrated efficacy in mitigating LPS-induced weight loss and anorexia, emphasizing its potential protective effects. The cytokine profiles revealed a consistent reduction in tumor necrosis factor α, highlighting the potent anti-inflammatory effects of palm^11^-PrRP31. The peptide also modulated key cytokines and chemokines in the plasma, liver and hypothalamus, reflecting its broad-spectrum anti-inflammatory properties. Palm^11^-PrRP31 also effectively attenuated the expression levels of Toll-like receptor 4 signaling components in the liver, suggesting its ability to suppress the activation of these pathways during LPS-induced inflammation. These anti-inflammatory effects were specific to palm^11^-PrRP31, whereas natural PrRP31 had a minimal impact. In conclusion, this study reveals the efficacy of palm^11^-PrRP31 in modulating LPS-induced inflammation, offering insights into its immunomodulatory properties. The ability of the peptide to suppress proinflammatory responses and attenuate relevant signaling pathways indicates its potential use as a therapeutic agent for inflammatory disorders.

## Introduction

Lipopolysaccharides (LPS) are the main structural components of the outer membrane in most gram-negative bacteria. These bacteria produce endotoxemia, which may cause septic shock. LPS can cause inflammation that stimulates the natural immune system. LPS also play a key role in the initiation of inflammation, which causes the release of inflammatory cytokines, reactive oxygen species and arachidonic acid metabolites. In addition, LPS-induced signaling and immune dysregulation are relevant in the pathophysiology of many diseases where endotoxemia is less severe, including neurodegenerative, metabolic and cardiovascular diseases (Faraj *et al.* 2017, Kar *et al.* 2022, Page *et al.* 2022).

In experimental settings, LPS administration in rats has been widely employed as a valuable substance model to study acute inflammatory responses and the underlying mechanisms. LPS induce an inflammatory response mainly by activating the Toll-like receptor 4 (TLR4) signaling pathway. TLR4, nuclear factor-kappa B (NF-κB) and mitogen-activated protein kinases (MAPKs) are understood to be the main signaling pathways involved in the inflammatory response (Akira & Takeda 2004). Among the MAPKs family members, extracellular signal-regulated kinase 1/2 (ERK 1/2), extracellular signal-regulated kinase 5 (ERK 5), c-Jun N-terminal kinases (JNK) and p38 mitogen-activated protein kinases (p38) are involved in the regulation of inflammation. Thus, inhibition of the TLR4, NFκB and MAPK signaling cascades could be a target for the treatment of inflammatory diseases (Holmes-McNary & Baldwin 2000, Baumgarten *et al.* 2001, Pearson *et al.* 2001, Lu *et al.* 2008, Wang *et al.* 2017).

Several studies have shown the anti-inflammatory properties of specific neuropeptides, highlighting their potential to attenuate the adverse effects of LPS-induced inflammation in experimental rat models (Dimitrijević *et al.* 2008, Yanay *et al.* 2015). It has been shown that peptides and neuropeptides regulating food intake could affect the immune response and could also be effective in the treatment of acute endotoxemia. Orexigenic neuropeptide Y or ghrelin or anorexigenic glucagon-like peptide-1 receptor agonists (Sachot *et al.* 2004, Dimitrijević *et al.* 2008, Yanay *et al.* 2015, Faim *et al.* 2019) were reported to act in this way.

Prolactin-releasing peptide (PrRP) is an anorexigenic neuropeptide that is produced by and acts in the brain. We recently designed PrRP31 analogs, palmitoylated at the N-terminus (palm-PrRP31) or at position 11 (palm^11^-PrRP31), which are more stable than the natural peptide and are able to act centrally after peripheral administration (Maletínská *et al.* 2015, Pražienková *et al.* 2017, Mráziková *et al.* 2021).

In aged Wistar Kyoto (WKY) rats with obesity induced by a high-fat diet, palm^11^-PrRP31 exhibited anti-obesity and glucose-lowering properties and reduced glucose intolerance (Mráziková *et al.* 2023). In addition, palm^11^-PrRP31 lowered blood glucose in Koletsky–spontaneously hypertensive obese (SHROB) rats, a model of metabolic syndrome (Mikulášková *et al.* 2018). In obese diabetic fatty (fa/fa) rats with leptin signaling disruption, palm^11^-PrRP31 displayed neuroprotective effects without anti-obesity or glucose-lowering effects (Mráziková *et al.* 2022).

Palmitoylated PrRP analogs undoubtedly show promise in the treatment of obesity and type 2 diabetes mellitus and exhibit neuroprotective effects in different rat and mouse models. The aim of this study was to evaluate the anti-inflammatory effect of palm^11^-PrRP31 and elucidate its potential mechanism in a rat model of acute LPS-induced inflammation.

A systemic inflammatory response was induced in WKY rats treated with the previously reported LPS doses (Kakizaki *et al.* 1999, Nonoguchi *et al.* 2022), and pretreatment with palm^11^-PrRP31 affected the inflammatory response.

## Materials and methods

### Chemicals

LPS from *Escherichia coli* O55:B5 were obtained from Sigma-Aldrich (Merck, Germany). The natural PrRP31 peptide and PrRP31, palmitoylated at position 11 (palm^11^-PrRP31) with the sequence SRTHRHSMEIK (N-γ-E(N-palmitoyl)) TPDINPAWYASRGIRPVGRF-NH_2_, were synthesized at the Institute of Organic Chemistry and Biochemistry of the Czech Academy of Sciences (IOCB CAS), Prague, Czech Republic, as previously described (Pražienková *et al.* 2017).

### Animals and experimental design

Eight-week-old male WKY rats were purchased from Charles River (Germany). The animals were maintained at a constant temperature of 22 ± 2°C under a 12 h light: 12 h darkness cycle, given free access to water and fed a standard rodent chow diet of ssniff R/M-H (ssniff Spezialdiäten GmbH, Germany) containing 33% protein, 9% fat and 58% carbohydrates. All procedures were performed in accordance with the ethical guidelines for work with animals as specified in the Act 246/1992 of the Czech Republic and approved by the Committee for Experiments with Laboratory Animals of the Czech Academy of Sciences.

#### Experimental approach

##### Experiment 1

**Figure 1:**
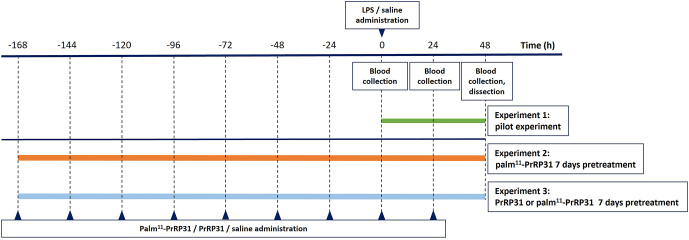
Design of the experiments involving WKY rats treated with LPS and the anorexigenic peptide palm^11^-PrRP31 or natural PrRP31. A full color version of this figure is available at https://doi.org/10.1530/JME-24-0090.

To optimize the experimental procedure, we conducted a pilot experiment consisting of 24 WKY rats divided randomly into four groups of six rats: a control group injected intraperitoneally (IP) with saline (saline IP) and three LPS groups injected with 1 mg/kg IP (LPS 1 mg/kg IP), 5 mg/kg IP (LPS 5 mg/kg IP) or 1 mg/kg intravenously (IV) (LPS 1 mg/kg IV). The doses 1 mg/kg (IP), 5 mg/kg (IP) and 1 mg/kg (IV) of LPS were selected according to the literature (García-Martínez *et al.* 1995, Hrupka & Langhans 2001, Ayaz *et al.* 2017, Yegla & Foster 2019, Alshehri & Imam 2021, Nonoguchi *et al.* 2022). The experimental design is shown in Fig. 1.

##### Experiment 2

In the next experiment, 24 WKY rats were randomly divided into four groups of six rats: a control group treated with saline for 7 days followed by saline (saline/saline), a group treated with saline for 7 days followed by LPS (saline/LPS), a group treated with palm^11^-PrRP31 for 7 days followed by saline (palm^11^-PrRP31/saline) and a group treated with palm^11^-PrRP31 for 7 days followed by LPS (palm^11^-PrRP31/LPS). The experimental design is shown in Fig. 1.

##### Experiment 3

In the last experiment, 18 WKY rats were randomly divided into three groups of six rats: a control group treated with saline for 7 days followed by saline administration (saline/saline), a group treated with saline for 7 days followed by LPS administration (saline/LPS) and a group treated with natural PrRP31 for 7 days followed by LPS administration (PrRP31/LPS). The experimental design is shown in Fig. 1.

All rats in Experiment 2 and Experiment 3 were injected IP with 1 mg/kg LPS, 5 mg/kg palm^11^-PrRP31 or 5 mg/kg PrRP31. Palm^11^-PrRP31 and PrRP31 were dissolved in saline. The dose of palm^11^-PrRP31 used in this study was chosen according to our previous studies (Holubová *et al.* 2016, Mikulášková *et al.* 2018, Mráziková *et al.* 2021, Mráziková *et al.* 2023) on the basis of an effective dose of peptides.

Body weight (BW) was measured every 24 h during saline or palm^11^-PrRP31 pretreatment and then 24 and 48 h after LPS or saline administration. Food intake was monitored 24 and 48 h after saline or LPS injection. Blood samples were collected 4, 24 and 48 h after saline or LPS administration. The rats were deeply anesthetized with pentobarbital (170 mg/kg BW, Sigma-Aldrich) 48 h after saline or LPS injection and then transcardially perfused with 10 mM ice-cold phosphate-buffered saline at a pH of 7.4 supplemented with heparin (10 U/mL, Zentiva, Czech Republic). Samples of liver tissue and hypothalamus were dissected and stored at −80°C until use.

### Tissue preparation for immunoblotting

The liver and hypothalamus samples were homogenized with a Qiagen tissue homogenizer (iBioTech a.s., Czech Republic) using 200 μL lysis buffer per sample (62.5 mM Tris–HCl buffer at pH 6.8, 1% deoxycholate, 1% Triton X-100, 50 mM NaF, 1 mM Na_3_VO_4_ and complete protease inhibitor (Roche Applied Science, Germany)). Each sample was placed in Eppendorf tubes containing one clean 5 mm Qiagen stainless steel bead in each tube. Homogenization was performed at 25 strokes/s at bursts of 30 s. The total homogenization time differed for the hypothalamus (2–3 bursts) and liver (3–4 bursts) tissue samples. All samples were kept on ice during tissue handling as follows: lysates were placed in new tubes on ice, sonicated for 1 min and centrifuged at 7000 *g* for 10 min. Supernatants were transferred to new tubes, and protein concentrations were determined via a Pierce BCA protein assay kit (Thermo Fisher Scientific, Inc., USA). The lysates were diluted to a final concentration of 1 g/L in Laemmli sample buffer (62.5 mM Tris–HCl at pH 6.8, 2% SDS, 10% glycerol, 0.01% bromophenol blue, 5% mercaptoethanol, 50 mM NaF and 1 mM Na_3_VO_4_) and stored at −20°C until use.

### Immunoblotting

The samples for immunoblotting were sonicated for 1 min and boiled at 100°C for an additional 2 min. Subsequently, 10 μg/10 μL of each sample was separated using SDS-PAGE (4–15% gradient). The following primary antibodies were obtained from Cell Signaling Technology (USA): protein kinase B (Akt), inhibitor of nuclear factor kappa B kinase subunit β (IKKβ), pIKKβ, JNK, pJNK, nuclear factor kappa B (NFκBp65), pNFκBp65, p38, pp38, phosphoinositide 3-kinase (PI3K) and pPI3K. The TLR4 primary antibody was obtained from Abcam (UK). We used a 1:1000 dilution of Tris-buffered saline (TBS) and Tween 20 (20 mM Tris, 136 mM NaCl and 0.1% Tween 20) with 5% milk.

The proteins were transferred onto a nitrocellulose membrane, blocked in 5% nonfat milk or BSA in TBS/Tween 20 buffer and incubated overnight with the corresponding primary antibody at 4°C. After incubation for 1 h with the IRDye 800CW goat anti-mouse IgG secondary antibody or the IRDye 680CW goat anti-rabbit IgG secondary antibody (both from iBioTech a.s., Czech Republic) at room temperature, the membranes were detected using a LI-COR device (LI-COR Biosciences, USA). The protein level was normalized to that of glyceraldehyde-3-phosphate dehydrogenase (Cell Signaling Technology, USA), which was used as the housekeeping protein.

### Detection of cytokines and chemokines

In plasma, liver and hypothalamus samples, the levels of TNFα and IL-6 were determined using an enzyme-linked immunosorbent assay (ELISA) kit (Thermo Fisher Scientific, USA) or a panel of interleukin-1β (IL-1β), IL-6, interleukin-10 (IL-10), TNFα, C–X–C motif chemokine ligand 10 (CXCL10) and C–C motif chemokine ligand 2 (CCL2). The concentrations were determined using a ProcartaPlex assay (Invitrogen, USA) using a MAGPIX System (Luminex Corporation, USA), according to the manufacturer’s instructions. EC_50_ values were calculated via the GraphPad Prism software (GraphPad Software, USA).

### Statistical analysis

The data are presented as mean ± S.E.M. Normality checks for data distribution were performed for each dataset. Statistical analysis was performed via *t* tests, one-way ANOVA followed by Bonferroni’s multiple comparison test or two-way ANOVA followed by Bonferroni’s multiple comparison test using the GraphPad Prism software.

## Results

### Experiment 1: IP administration of 1 mg/kg LPS induces the most significant changes

**Figure 2:**
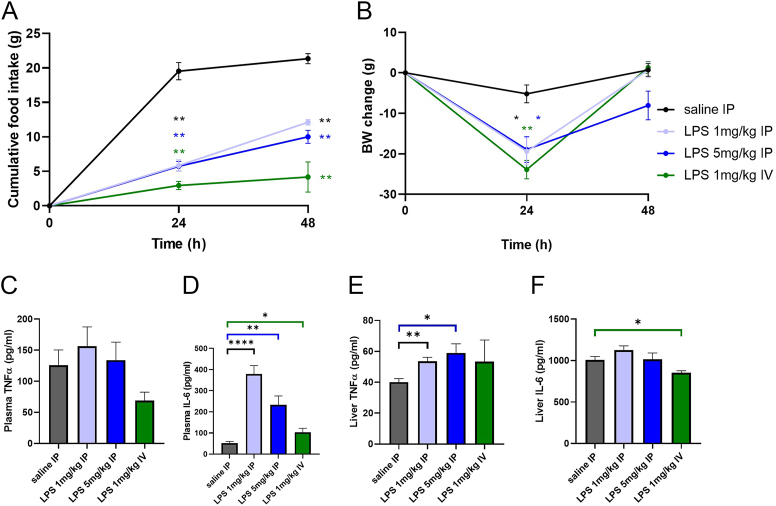
Experiment 1 shows the effects of LPS on BW, food intake and proinflammatory cytokines. LPS 1 mg/kg IP, LPS 5 mg/kg IP and LPS 1 mg/kg IV represent the different doses and routes of LPS administration. (A) Food intake and (B) BW changes were measured 24 and 48 h after LPS administration. Plasma levels of (C) TNFα and (D) IL-6 were measured 4 h after LPS administration. The levels of (E) TNFα and (F) IL-6 in the liver were measured 48 h after LPS administration; cytokines were measured via ELISA. The data are presented as mean ± S.E.M. Statistical analysis was performed via two-way ANOVA with Bonferroni *post hoc* correction or the *t* test. BW, body weight; IL-6, interleukin-6; LPS, lipopolysaccharides; TNFα, tumor necrosis factor α. *P < 0.05, **P < 0.01, and ****P < 0.0001 for the LPS 1 mg/kg IP group vs the saline IP group; *P < 0.05 and **P < 0.01 for the LPS 5 mg/kg IP group vs the saline IP group; *P < 0.05 and **P < 0.01 for the LPS 1 mg/kg IV group vs the saline IP group (*n* = 5–6). A full color version of this figure is available at https://doi.org/10.1530/JME-24-0090.

The results of Experiment 1 are shown in Fig. 2.

Food intake and BW were measured 24 and 48 h after LPS administration. Food intake (Fig. 2A) and BW (Fig. 2B) significantly decreased after all doses and administrations of LPS. We observed no significant changes in plasma TNFα (Fig. 2C) after 4 h in any mode of LPS treatment. However, the plasma IL-6 concentration significantly increased at 4 h after treatment with LPS (Fig. 2D); the effects of IP administration of 1 mg/kg LPS were the most significant. In the liver, 48 h after LPS administration, the level of TNFα significantly increased after IP administration at both LPS doses (Fig. 2E). We found no significant changes in the IL-6 concentration in the liver after the IP administration of LPS. However, following IV administration of LPS, the concentration of IL-6 in the 1 mg/kg LPS IV group was significantly lower than that in the saline group. As shown in Fig. 2, IP administration of LPS at a dose of 1 mg/kg was associated with the most significant changes. Therefore, this route of administration was selected for subsequent experiments.

### Experiment 2: palm^11^-PrRP31 prevents the production of proinflammatory cytokines and chemokines in plasma, liver and hypothalamus samples

**Figure 3:**
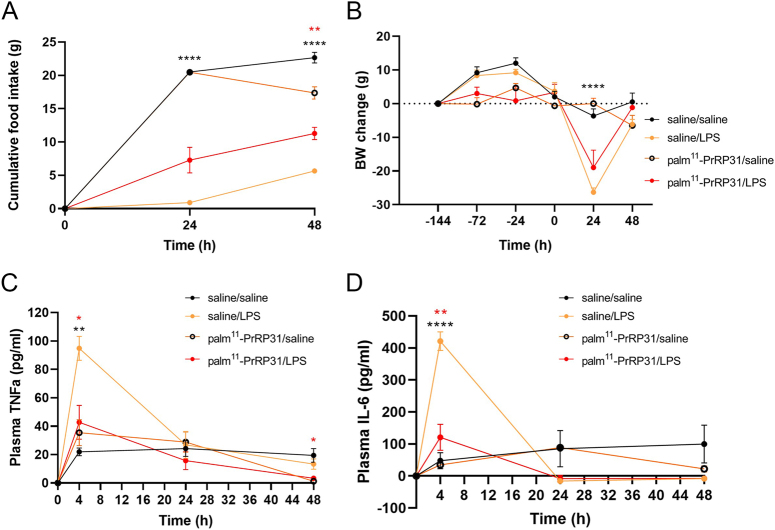
Experiment 2 shows the effects of palm^11^-PrRP31 pretreatment on food intake, BW and proinflammatory cytokines in LPS-treated rats. Palm^11^-PrRP31/LPS indicates the group pretreated with palm^11^-PrRP31 for 7 days before IP administration of 1 mg/kg LPS. LPS were administered at time point 0. (A) Food intake was measured 24 and 48 h after LPS administration. (B) Changes in BW were measured every day during saline or palm^11^-PrRP31 pretreatment. Plasma levels of (C) TNFα and (D) IL-6 were measured 4, 24 and 48 h after LPS administration. TNFα and IL-6 were measured via ELISA. The data are presented as mean ± S.E.M. Statistical analysis was performed via two-way ANOVA with Bonferroni *post hoc* correction or the *t* test. BW, body weight; IL-6, interleukin-6; LPS, lipopolysaccharides; TNFα, tumor necrosis factor α. Significance is denoted as **P < 0.01 and ****P < 0.0001 for saline/LPS vs saline/saline and *P < 0.05 and **P < 0.01 for palm^11^-PrRP31/LPS vs saline/LPS (*n* = 5–6). A full color version of this figure is available at https://doi.org/10.1530/JME-24-0090.

**Figure 4:**
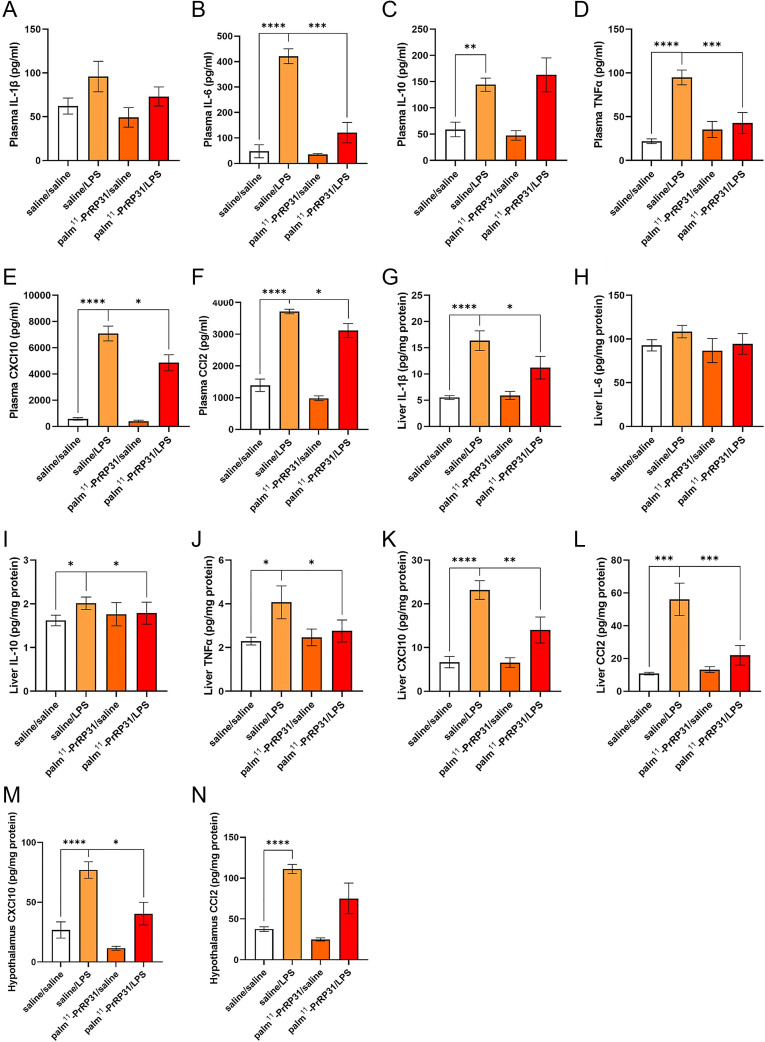
Experiment 2 shows the effects of palm^11^-PrRP31 pretreatment on the levels of inflammatory cytokines and chemokines in the plasma, liver and hypothalamus of LPS-treated rats. Palm^11^-PrRP31/LPS represents the group pretreated with palm^11^-PrRP31 for 7 days before LPS administration. (A), (B), (C) and (D) Plasma cytokines and (E) and (F) plasma chemokines were measured 4 h after LPS administration via a ProcartaPlex assay using the MAGPIX System. (G), (H), (I) and (J) Liver cytokines, (K) and (L) liver chemokines and (M) and (N) hypothalamus chemokines were measured 48 h after LPS administration via the ProcartaPlex assay using the MAGPIX System. The data are presented as mean ± S.E.M. Statistical analysis was performed via one-way ANOVA with Bonferroni *post hoc* correction. BW, body weight; CCl2, chemokine C–C motif ligand 2; CXCl10, C–X–C motif chemokine ligand 10; IL-6, interleukin-6; LPS, lipopolysaccharides; TNFα, tumor necrosis factor α. *P < 0.05, **P < 0.01, ***P < 0.001 and ****P < 0.0001 (*n* = 5–6). A full color version of this figure is available at https://doi.org/10.1530/JME-24-0090.

**Figure 5:**
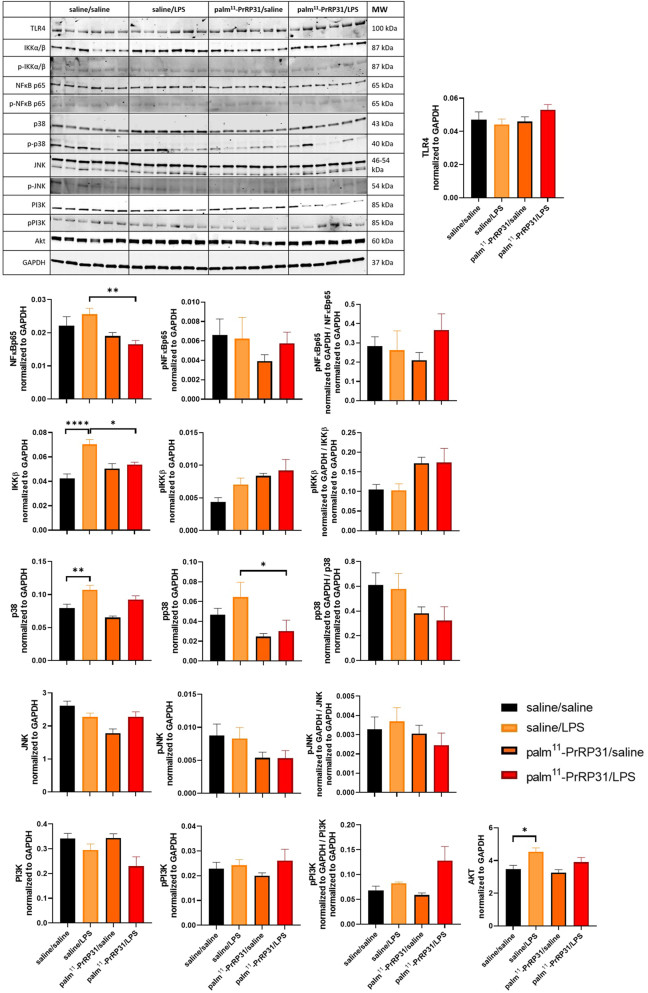
Experiment 2 shows the protein and phosphorylated protein levels of TLR4, NFkBp65 and MAPK in the liver. Protein and phosphorylated protein levels of TLR4, IKKb, NFκBp65, p38, JNK, PI3K and Akt were determined by western blotting 48 h after LPS or saline administration. The data are presented as mean ± S.E.M. Statistical analysis was performed via t tests or one-way ANOVA with Bonferroni *post hoc* correction. LPS, lipopolysaccharides; TLR4, Toll-like receptor 4; IKKβ, inhibitor of nuclear factor kappa B kinase subunit β; NFκBp65, nuclear factor kappa-B; p38, p38 mitogen-activated protein kinase; JNK, c-Jun N-terminal kinases; PI3K, phosphoinositide 3-kinase; Akt, protein kinase B. *P < 0.05, **P < 0.01, ***P < 0.001 and ****P < 0.0001 (*n* = 5–6). A full color version of this figure is available at https://doi.org/10.1530/JME-24-0090.

The results of Experiment 2 are shown in Figs 3, 4, 5.

Cumulative food intake was monitored 24 and 48 h after LPS or saline administration. BW was monitored every 24 h during pretreatment with saline or palm^11^-PrRP31 for 7 days (5 mg/kg, IP) and 24 and 48 h after LPS or saline administration. Compared with that in the saline/saline group, the cumulative food intake significantly decreased in the saline/LPS group. Compared with the saline/LPS treatment, palm^11^-PrRP31 treatment significantly increased the cumulative food intake in the palm^11^-PrRP31/LPS group (Fig. 3A). The change in BW was significantly lower in the saline/LPS group than in the saline/saline group. We observed a protective trend in the palm^11^-PrRP31/LPS group after palm^11^-PrRP31 pretreatment before the decrease in BW caused by LPS compared with that in the saline/LPS group 24 h after LPS administration (Fig. 3B). Figure 2C and D show the plasma profiles of the proinflammatory cytokines TNFα and IL-6 at 4, 24 and 48 h following the administration of LPS or saline. The most notable increase in TNFα occurred 4 h after LPS administration in the saline/LPS group. Across all time points in the plasma TNFα profile (Fig. 3C), palm^11^-PrRP31 significantly decreased the TNFα concentration. Compared with that in the saline/saline group, a significant increase in IL-6 concentration was observed 4 h after LPS administration in the saline/LPS group, and compared with the saline/LPS treatment, palm^11^-PrRP31/LPS treatment led to a significant decrease in IL-6 levels 4 h after LPS administration in the palm^11^-PrRP31/LPS group (Fig. 3D).

The cytokine and chemokine concentrations in the plasma measured 4 h after LPS or saline administration are shown in Fig. 4. Compared with those in the saline/saline control group, the production of proinflammatory cytokines, including IL-6 and TNFα (Fig. 4A), and the proinflammatory chemokines CXCL10 and CCL2 (Fig. 4B), significantly increased in the saline/LPS group. We observed an increase in IL-1β levels (Fig. 4A) in the saline/LPS group compared with the saline/saline group. On the other hand, the levels of these cytokines (IL-6 and TNFα) and chemokines (CXCL10 and CCL2) significantly decreased in the group treated with palm^11^-PrRP31/LPS. The anti-inflammatory cytokine IL-10 was significantly greater in the saline/LPS group than in the saline/saline group. We found no significant differences between the palm^11^-PrRP31/LPS and saline/LPS groups (Fig. 4A). Palm^11^-PrRP31 had no effect on proinflammatory or anti-inflammatory cytokines or chemokines in the palm^11^-PrRP31/saline group compared with those in the saline/saline group.

In the liver, the concentrations of proinflammatory cytokines, including IL-1β and TNFα (Fig. 4C), were significantly greater in the saline/LPS group than in the saline/saline group. Compared with the saline/LPS treatment, palm^11^-PrRP31 treatment significantly decreased the levels of these proinflammatory cytokines in the palm^11^-PrRP31/LPS group. Compared with that in the saline/saline group, the level of the anti-inflammatory cytokine IL-10 in the liver significantly increased in the saline/LPS group (Fig. 4C). However, the level of IL-10 in the palm^11^-PrRP31/LPS group was significantly lower than that in the saline/LPS group. Compared with those in the saline/saline group, the levels of the chemokines CXCL10 and CCL2 (Fig. 4D) significantly increased in the saline/LPS group, whereas treatment with palm^11^-PrRP31 significantly decreased the levels of these chemokines. There were no changes in any of the proinflammatory or anti-inflammatory cytokines or chemokines induced by palm11-PrRP31-in the palm^11^-PrRP31/saline group compared with those in the saline/saline group.

The levels of the chemokines CXCL10 and CCL2 in the hypothalamus were significantly greater in the saline/LPS group than in the saline/saline group (Fig. 4E). Treatment with palm^11^-PrRP31 significantly decreased the level of CXCL10. However, there were no significant changes in CCL2 in the palm^11^-PrRP31/LPS group compared with the saline/LPS group. The levels of these chemokines in the palm^11^-PrRP31/saline group did not differ from those in the saline/saline group.

### Experiment 2: palm^11^-PrRP31 attenuates the protein levels of TLR4, NFkBp65 and MAPK signaling members in the liver

LPS treatment increased IKKβ, p38 and AKT protein levels in the liver. Palm^11^-PrRP31 pretreatment before LPS intervention attenuated liver IKKβ and pp38. Although the LPS-induced increase in NF-κBp65 level did not reach significance, palm^11^-PrRP31 pretreatment significantly decreased the NF-κBp65 protein level in the liver. The protein levels of JNK and PI3K and their phosphorylated forms in the liver were not affected by LPS treatment, and palm^11^-PrRP31 pretreatment had no effect on these proteins (Fig. 5).

### Experiment 3: natural PrRP31 has no effect on food intake, BW or cytokine production

**Figure 6:**
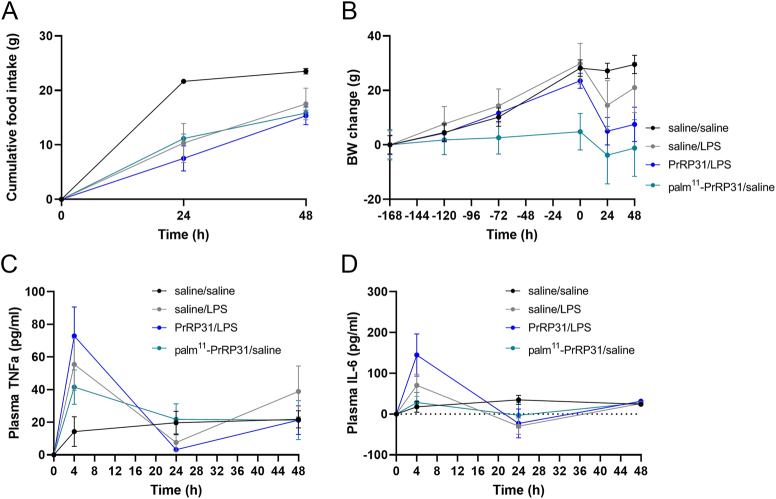
Experiment 3 shows the effects of PrRP31 pretreatment on BW, food intake and proinflammatory cytokines in LPS-treated rats. PrRP31/LPS represents the group pretreated with PrRP31 for 7 days before LPS administration. (A) Food intake was measured 24 and 48 h after LPS administration. (B) Changes in BW were measured every day during saline or PrRP31 pretreatment. Plasma levels of (C) TNFα and (D) IL-6 were measured 4, 24 and 48 h after LPS administration. TNFα and IL-6 were measured via ELISA. The data are presented as mean ± S.E.M. Statistical analysis was performed via two-way ANOVA with Bonferroni *post hoc* correction or the *t* test. BW, body weight; IL-6, interleukin-6; LPS, lipopolysaccharides; TNFα, tumor necrosis factor α. A full color version of this figure is available at https://doi.org/10.1530/JME-24-0090.

The results of Experiment 3 are shown in Fig. 6.

Pretreatment with the natural peptide PrRP31 affected neither cumulative food intake nor BW changes in the LPS-treated rats. Similarly, pretreatment had no effect on LPS-induced TNFα or IL-6 in the plasma.

## Discussion

This is the first study to investigate the immunomodulatory effects of the food intake-regulating peptide palm^11^-PrRP31 on LPS-induced inflammation in rats. Our findings revealed significant alterations in cumulative food intake, BW and concentrations of proinflammatory cytokines and chemokines in the plasma, liver and hypothalamus after palm^11^-PrRP31 pretreatment. These results underscore the potential of palm^11^-PrRP31 in suppressing the inflammatory response elicited by LPS and its impact on key physiological parameters.

In this study, we used inbred Wistar Kyoto rats, which are often used as controls in studies of genetic hypertension and are also known to be more sensitive to stress compared with other strains, such as Lewis and Sprague–Dawley rats. However, WKY rats, along with other rat models, were used in several studies of inflammation induced by LPS administration (Devaux *et al.* 2001, Baranowska-Bik *et al.* 2006, Chen *et al.* 2017). Moreover, O’Mahony and coworkers compared the effects of LPS in WKY and Sprague–Dawley rats (O’Mahony *et al.* 2013), showing that peripheral immune response to LPS was similar in both strains; the central neurochemical response was attenuated in WKY rats. The main reason to use WKY rats in this study was based on our previously published results (Cermakova *et al.* 2019, Mráziková *et al.* 2023), in which we observed a significant anti-obesity effect of palmitoylated-PrRP in diet-induced obese WKY rats.

In Experiment 1, we strategically assessed different doses and administration routes of LPS to determine the most effective conditions for inducing inflammation in rats. We chose IP administration at a dose of 1 mg/kg LPS after observing significant reductions in BW and food intake compared with the other doses and administration routes we monitored. Moreover, this LPS concentration significantly increased the plasma IL-6 levels while maintaining a consistent increase in TNFα, reinforcing its ability to elicit a robust inflammatory response.

The observed decrease in BW in the saline/LPS group in Experiment 2 aligns with previous studies, indicating the impact of LPS-induced inflammation on morphometric parameters (Hrupka & Langhans 2001, Sachot *et al.* 2004, Zhong *et al.* 2018, Kurniawan *et al.* 2023).

Pretreatment with palm^11^-PrRP31 did not significantly influence the change in BW.

We previously reported that chronic administration of palm^11^-PrRP31 did not affect the BW of nonobese lean rats or mice fed a standard diet (Holubova *et al.* 2019). Therefore, the beneficial effect of palm^11^-PrRP31 on LPS-induced anorexia is caused by its impact on acute inflammation and not by its anorexigenic effect, which occurs only in obese but not in lean individuals.

The activated signaling pathways control the release of inflammatory cytokines, which play crucial roles in the inflammatory response. Interleukins such as IL-1β, IL-6 and TNFα have been identified as markers of LPS-induced acute inflammation. Our temporal analysis of the TNFα and IL-6 profiles revealed significant changes following LPS administration. The pronounced increase in TNFα at 4 h in the saline/LPS group is consistent with the acute phase response triggered by LPS (Pfeffer *et al.* 1993, Li *et al.* 2008, Schulte-Michels *et al.* 2016).

Importantly, palm^11^-PrRP31 consistently reduced TNFα levels at all time points, underscoring its potent anti-inflammatory effects. Similarly, the significant decrease in IL-6 levels at 4 h after LPS administration in the palm^11^-PrRP31/LPS group points to the ability of the peptides to modulate cytokine production during the early stages of inflammation.

In Experiment 2, we demonstrated that IP administration of LPS increased the expression of IL-1β, IL-6, IL-10, CXCL10 and CCL2 in the periphery 4 h (plasma) and 48 h (liver) after LPS administration, which aligns with previous studies (Davis *et al.* 2017, Cao *et al.* 2021, Myers *et al.* 2023, Papaioannou *et al.* 2023). Treatment with palm^11^-PrRP31 effectively suppressed the production of proinflammatory cytokines (IL-6 and TNFα) and chemokines (CXCL10 and CCL2), highlighting its broad-spectrum anti-inflammatory properties. Notably, palm^11^-PrRP31 had no significant effect on anti-inflammatory IL-10 levels in plasma, indicating targeted modulation of proinflammatory pathways. In the liver, palm^11^-PrRP31 significantly attenuated the LPS-induced increase in the levels of proinflammatory cytokines (IL-1β and TNFα) and chemokines (CXCL10 and CCL2). Interestingly, palm^11^-PrRP31 had a differential effect on IL-10 levels, reducing its concentration in the liver without affecting its plasma levels. Our recent results are not able to explain why palm^11^-PrRP31 affects only the proinflammatory cytokine TNF-α and IL-6 but not the anti-inflammatory cytokine IL-10. However, further studies are needed to elucidate the precise mechanism by which palm^11^-PrRP31 affects cytokine production and/or cytokine release.

Previous studies have documented the anti-inflammatory effects of IL-10, but it also exerts immunomodulatory effects on immune responses. Because of its diverse functions, which include the promotion and suppression of inflammation and the protection and regeneration of tissue, the IL-10 cytokine has a highly complex effect on the organism. In addition, it plays crucial roles in bolstering the host defense against diverse pathogens, reinstating the host to an immune quiescent state and maintaining tissue homeostasis following inflammatory responses (Jung *et al.* 2013, Ouyang & O’Garra 2019). In our study, treatment with palm^11^-PrRP31 failed to significantly alter the increase in IL-10 in plasma. As such, further studies are needed to elucidate the mechanism of action.

We were also interested in the effects of LPS on the expression of cytokines and chemokines in the brain, particularly on neuroinflammation, as previously shown in the literature (Davis *et al.* 2017, Myers *et al.* 2023). We were unable to detect the cytokines IL-1β, IL-6, IL-10 and TNFα 48 h after LPS administration, a finding that is consistent with the literature (Alperina *et al.* 2019, Myers *et al.* 2023). We found that the chemokines CXCL10 and CCL2 were elevated in the hypothalamus 48 h after LPS administration in the saline/LPS group and that this same treatment effectively mitigated the LPS-induced increase in CXCL10 level in the palm^11^-PrRP31/LPS group, underscoring its potential to modulate neuroinflammatory responses.

The attenuation of cytokine and chemokine production reflects the ability of palm^11^-PrRP31 to modulate systemic and tissue-specific inflammatory responses, underscoring its potential therapeutic relevance in mitigating cytokine storm-related pathologies. In our study of the anti-inflammatory properties of palm^11^-PrRP31, we conducted a detailed examination of protein expression levels in the liver, focusing on key components of the TLR4 signaling pathway, including TLR4 itself, IKKβ, NFκBp65, p38, PI3K, Akt and their phosphorylated forms.

In the liver, LPS significantly increased IKKβ, p38 and Akt protein levels, whereas NF-κBp65 only tended to increase. Palm^11^-PrRP31 dampened the effect of LPS and attenuated the protein expression of NF-κBp65, IKKβ and pp38 in the liver.

These findings suggest that palm^11^-PrRP31 exerts suppressive effects on the activation of the TLR4 signaling pathway during LPS-induced inflammation, which corresponds with the literature (Wang *et al.* 2017, Doğanyiğit *et al.* 2020, Khan *et al.* 2021). On the other hand, there were no significant differences in the levels of the PI3K protein or its phosphorylated form between the experimental groups. However, Akt protein levels were significantly elevated in the saline/LPS group compared with those in the saline/saline group; phosphorylated forms in the liver samples from Experiment 2 were not measurable. These findings indicate the potential activation of the PI3K/Akt pathway in response to LPS, which is consistent with its known role in cellular survival and inflammatory responses (Zhang *et al.* 2007, Xu *et al.* 2010).

**Figure 7:**
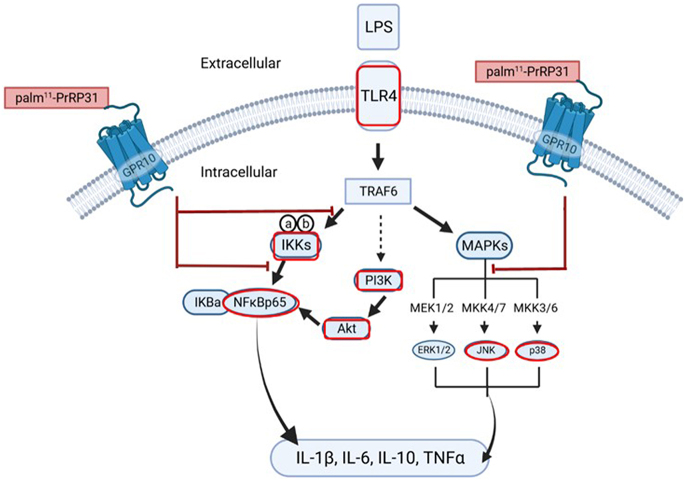
Schematic representation of a possible palm^11^-PrRP31 signaling cascade during the suppression of the LPS-induced inflammatory response in the liver. The black arrows indicate the progressive course of the inflammatory response induced by LPS. The red inverted-T symbols indicate the possible inhibitory effects of palm^11^-PrRP31. ERK 1/2, extracellular signal-regulated kinase 1/2; IKKβ, inhibitor of nuclear factor kappa B kinase subunit β; IL, interleukin; JNK, c-Jun N-terminal kinases; LPS, lipopolysaccharides; MAPKs, mitogen-activated protein kinases; NFκBp65, nuclear factor kappa B; p38, p38 mitogen-activated protein kinases; TLR4, Toll-like receptor 4; TNFα, tumor necrosis factor α; TRAF6, TNF receptor-associated factor 6. The parameters measured in this study are in red circles. A full color version of this figure is available at https://doi.org/10.1530/JME-24-0090.

The downregulation of these crucial inflammatory signaling components demonstrates the regulatory role of palm^11^-PrRP31 in modulating inflammatory pathways. These findings increase our understanding of the molecular mechanisms by which palm^11^-PrRP31 exerts its anti-inflammatory effects in the context of LPS-induced inflammation (Fig. 7).

In contrast, the preventive peripheral administration of natural PrRP31 in Experiment 3 had minimal effects on food intake, BW and cytokine production. These results suggest that peripheral administration of natural PrRP31 does not exert anti-inflammatory effects. The only anti-inflammatory effects we observed were specific to the palm^11^-PrRP31 peptide in Experiment 2, emphasizing the importance of this modified structure in enhancing peptide efficacy. Thus, lipidization was introduced to improve PrRP stability (which is known from our previous studies (Maletínská *et al.* 2015, Strnadova *et al.* 2023)), which could be one of the reasons why lipidized PrRP has shown anti-inflammatory effects and natural PrRP has not. Our previous studies revealed that lipidized PrRP analogs, such as palm^11^-PrRP31, exhibit central anorexigenic effects after peripheral administration, leading to a dose-dependent reduction in food intake in mice (Maletínská *et al.* 2015, Pražienková *et al.* 2017, Pirník *et al.* 2021). Neuronal activity, reflected by increased c-Fos expression in areas of the brain related to food intake regulation, is notably elevated after subcutaneous application of lipidized PrRP31 analogs but not after natural PrRP31 administration (Maletínská *et al.* 2015, Pražienková *et al.* 2017, Pirník *et al.* 2018). While the present study demonstrated the anti-inflammatory potential of palm^11^-PrRP31, further investigations are warranted to elucidate the underlying mechanism at play and any potential long-term effects. Assessing the impact of palm^11^-PrRP31 on other inflammatory mediators and its potential role in chronic inflammatory conditions is likely to provide a more comprehensive understanding of its therapeutic utility.

In summary, the results of our study show that palm^11^-PrRP31 is adept at modulating LPS-induced inflammation, highlighting its potential as a therapeutic agent. Palm^11^-PrRP31 exhibited anti-inflammatory effects by suppressing cytokine and chemokine production, attenuating the TLR4 signaling pathway and improving physiological parameters. These results provide valuable insights into the immunomodulatory properties of palm^11^-PrRP31, paving the way for future investigations and potential applications in inflammatory disorders.

## Declaration of interest

Lenka Maletinska is a Senior Editor of the *Journal of Molecular Endocrinology*. Lenka Maletinska was not involved in the review or editorial process for this paper, on which she is listed as an author.

## Funding

This work was supported by the National Institute for Metabolic and Cardiovascular Disease Research (EXCELES, LX22NP05104), a project funded by the European Union’s NextGenerationEU program. This work was also supported by the Czech Academy of Scienceshttps://doi.org/10.13039/501100004240 (RVO:61388963 and RVO:67985823).

## Author contribution statement

LM, JK, LM and SH contributed to the conception and design of the study. LM, JK, LM, SH and AS performed the experiments and collected the data. The first draft of the manuscript was written by LM. All authors commented on, reviewed and approved the final manuscript.

## Data availability

Enquiries about data availability should be directed to the corresponding author.

## Ethical approval

All procedures were performed in accordance with the ethical guidelines for work with animals as specified in the Act 246/1992 of the Czech Republic and approved by the Committee for Experiments with Laboratory Animals of the Czech Academy of Sciences (project no. 96/2020). The study is reported in accordance with ARRIVE guidelines.
